# Mini Review on Flexible and Wearable Electronics for Monitoring Human Health Information

**DOI:** 10.1186/s11671-019-3084-x

**Published:** 2019-08-01

**Authors:** Yiding Gu, Ting Zhang, Hao Chen, Feng Wang, Yueming Pu, Chunming Gao, Shibin Li

**Affiliations:** School of Optoelectronic Science and Engineering, University of Electronic Science and Technology of China, Chengdu, 610054 China

**Keywords:** Flexible sensors, Health monitoring, Force sensors, Temperature sensors, Physiological biochemical sensors, Multifunctional sensors

## Abstract

The application potential of wearable electronics in the healthcare field has been of great interest over the past several decades. Flexible and wearable devices based on skin-friendly soft elastic materials can be snugly attached to the surface of human skin, so that a series of vital health information such as wrist pulse, body temperature, and blood glucose can be extracted and analyzed to help the patient maintain physical fitness. Here, we outlined the most common types of wearable electronics for monitoring human health information, including force sensors, temperature sensors, physiological biochemical sensors, and multifunctional sensors. Their general working principles and structural innovations are reviewed. Then, we discussed two functional modules that make the wearable sensors more applicable in real life—self-powered module and signal processing module. The challenges and future research directions are also proposed to develop wearable electronics for monitoring human health information.

## Introduction

Since the 1950s, the rise of silicon-based semiconductor technology has greatly promoted the development of the information technology industry, making people’s lives change dramatically. However, with the acceleration of the world informationization and the development of the Internet of Things (IoT), conventional silicon-based electronics with high Young’s modulus are facing new challenges. Over the past few decades, flexible and wearable electronics have drawn increasing interest and become a hot topic in the science world. In contrast with rigid silicon-based electronic devices, flexible electronics exhibit many unique superior characteristics, such as high flexibility, ultralight weight, and conformality, which enable flexible and wearable electronics to be used in a wider range of application.

In particular, there has been a growing interest in flexible and wearable medical device for regular and continuous monitoring human health information. New devices are being invented to continuously monitor vital signs as comfortably as possible. These wearable medical electronic devices can measure various health indicators such as heart rate, pulse, body temperature, blood glucose, etc. noninvasively in real time by simply attaching them to the human body surface. Real-time monitoring of vital signs can alert users and health care providers to further medical care when an individual’s physical health indicators are abnormal, avoiding the situation where the best treatment time is missed. Also, flexible electronics can be deformed at will and detect various signals with extremely high sensitivity, thus can be used in artificial electronic skin, motion detection, telemedicine, and in-home healthcare. There is no doubt that next-generation flexible and wearable electronics will lead to a revolution in human way of life.

**Fig. 1:**
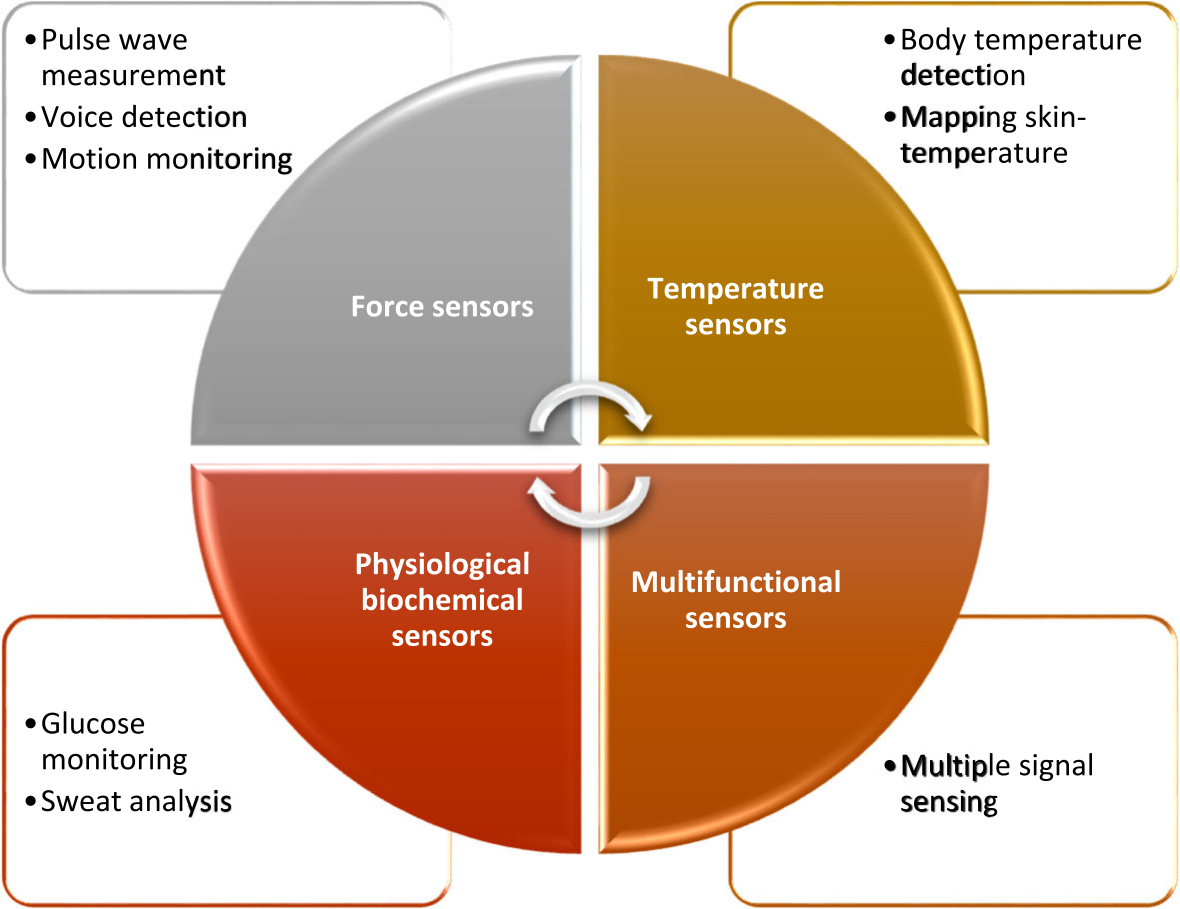
A visual summary of recent development of wearable electronics for monitoring human health information

Considerable efforts have been devoted to the production and development of wearable electronics and exciting advancements have been made in new materials, new process, and sensing mechanism during the past few years. As shown in Fig. 1, this review paper focuses on the development of wearable electronics for monitoring human health information, discussing their general working principles by citing some successful examples. In Section 2, we introduce force sensors for measuring body surface micro-strain caused by hemokinesis and human activity. Especially those microstructured stress or pressure sensors have ultra-high sensitivity and can be used to detect the pulse [1, 2], the voice [3], and the human motion [4]. In Section 3, temperature sensors for detecting and mapping skin temperature are reviewed. For temperature sensors, we focus on some solutions for enhancing the stretchability and decoupling strain interference from temperature effects. Besides physical signals, biological signals are also generated by human body’s normal activity. Physiological biochemical sensors for monitoring physiological biomarkers are describes in Section 4. In Section 5, we describe some multifunctional sensors that integrated multiple sensitive elements to perform simultaneous multi-channel signal detection. In order to truly realize the independent operation of wearable electronics, some practical functional modules like self-powered component and data processing module are necessary, which are briefly reviewed in Section 6. Finally, we summarize the developments of flexible and wearable electronics for monitoring human health information in recent years and prospect the perspective of flexible and wearable electronics for monitoring human health information.

## Flexible Force Sensors

The force sensor is a sensing device that can detect the values of mechanical forces such as tension, pressure, torque, stress, and strain and convert them into electrical signals. The various physical stimuli generated by regular physiological activity of human body contain many important health information, for instance, heart beat rate, muscle movement, respiration rate, and blood pressure. Most of traditional force sensors are bulky and heavy because they are mostly based on metal and semiconductor materials, and they are not applicable to wearable electronics for monitoring vital signs of human body due to their greatly limited portability and flexibility. Compared with traditional force sensors, flexible force sensors using plastic and elastomeric substrates have a series of advantages, such as better biocompatibility, stretchability, transparency, wearability, and capability of continuous detection. We shall discuss below that the flexible force sensors can be divided into resistivity sensors, capacitive sensors, and piezoelectric sensors.

### Resistive Force Sensors

A resistive sensor is a sensor that converts the change in resistance of sensitive materials caused by an external stimulus into an electrical signal output. The active materials of flexible resistive force sensors are generally elastomer composites formed by incorporating conductive fillers, such as graphene [5, 6], carbon nanotubes (CNTs) [7–10, 11], metallic thin film, nanowires, particles [12–14], and conductive polymers [15] into elastomers (e.g., PDMS, PU, SEBS). The resistance change of the sensor is mainly caused by the following three factors: (1) changes in the geometry of sensitive elements [15], (2) the change of the gap between nanoparticles or nanowires [5–10, 13, 14], and (3) changes in contact resistance between different layers of materials [12, 11]. Piezoresistive sensors have received widespread attention due to their low power consumption, simple manufacturing processes, and wide application [16].

**Fig. 2:**
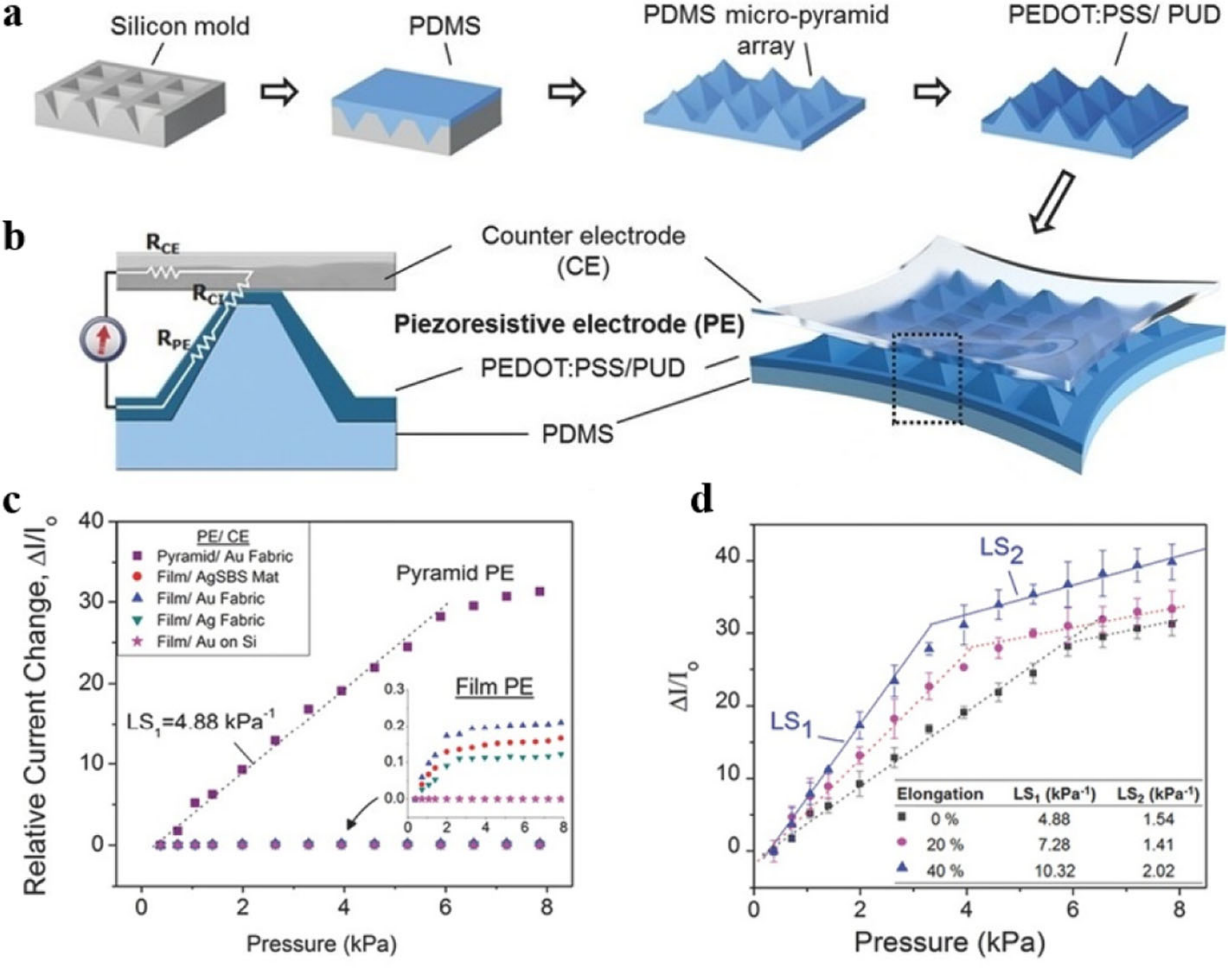
**a** Fabrication process of micropyramid PDMS array. **b** Schematic of sensing principle of the sensor with micropyramid structure under external force. **c** Improved sensitivity of pyramid sensors compared with unstructured sensors. **d** Linear pressure responses of micropyramid sensors when stretched. Adapted with permission from ref. 10. Copyright 2014 John Wiley and Sons

The utilization of substrates with microstructure surface offers an effective way to fabricate highly sensitive piezoresistive force sensors. As shown in Fig. 2a, b, Choong et al. [15] reported a flexible piezoresistive sensor by employing micropyramid polydimethylsiloxane (PDMS) array to enhance the pressure sensitivity of the sensor. This work proved that using micropyramid substrate can maximize the geometry change of the conductive electrode induced by pressure or stretching, significantly improving the sensitivity (Fig. 2c). As can be seen from Fig. 2d, the sensor has a good linear response to pressure. However, the fabrication of micropyramid structure was based on Si mold, which suffered from a complicated fabrication process and high cost [1, 3]. Wang et al. [1] used a piece of delicate silk scarf as the mold to fabricate micro-patterned PDMS substrate. In their work, a free-standing single-walled carbon nanotubes (SWCNTs) ultrathin film was transferred on the micro-patterned surface and the sensor was constructed by placing two layers of SWCNTs/PDMS films face-to-face. The sensor with surface microstructure prepared by using silk as template to prepare demonstrated high sensitivity, fast response time, great stability, ultralow detection limit, and excellent sensing performance in voice recognition and pulse detection in real time. In addition, Su et al. [17] reported PDMS thin film with an irregular pattern of microdomain using mimosa leaves. Wei et al. [18] produced microdome-structured PDMS films using ground glass substrates. These efforts provided simple and low-cost methods to fabricate large-area thin film substrate with microstructure and acquired good results in improving sensitivity of piezoresistive sensors. Inherently microstructured flexible materials, for instance papers [4], textile [19], plants, and plant-derived biomaterials [20, 21], have drawn a wide range of interest to be used as substrate. Tao et al. [4] reported graphene/paper-based pressure sensors for detecting human-activity. They mixed the tissue paper with the graphene oxide (GO) solution to obtain a GO paper. After heating in the drying oven for several hours, the GO paper was reduced to give an rGO/paper conductive composite. The sensitivity of the paper-based sensor in the pressure range of 0–20 kPa varies with the number of layers of tissue papers. The eight-layer sensor achieves a maximum sensitivity of 17.2 kPa^−1^ in the range of 0–2 kPa. The graphene/paper-based pressure sensor demonstrated great potential in monitoring wrist pulse, breathing, speaking, and motion states. In addition, Yang et al. [19] prepared a wearable strain sensor by reducing GO sheets to graphene sheets thermally on a polyester fabric substrate. The fabric substrate with interwoven structure rendered the sensor some special response characteristics, including ultra-high negative resistance-strain coefficient and unique direction sensitivity. The as-prepared textile strain sensor could be perfectly integrated with clothing for real-time monitoring human motion such as pulse, mouth motion, facial expression, and so on.

**Fig. 3:**
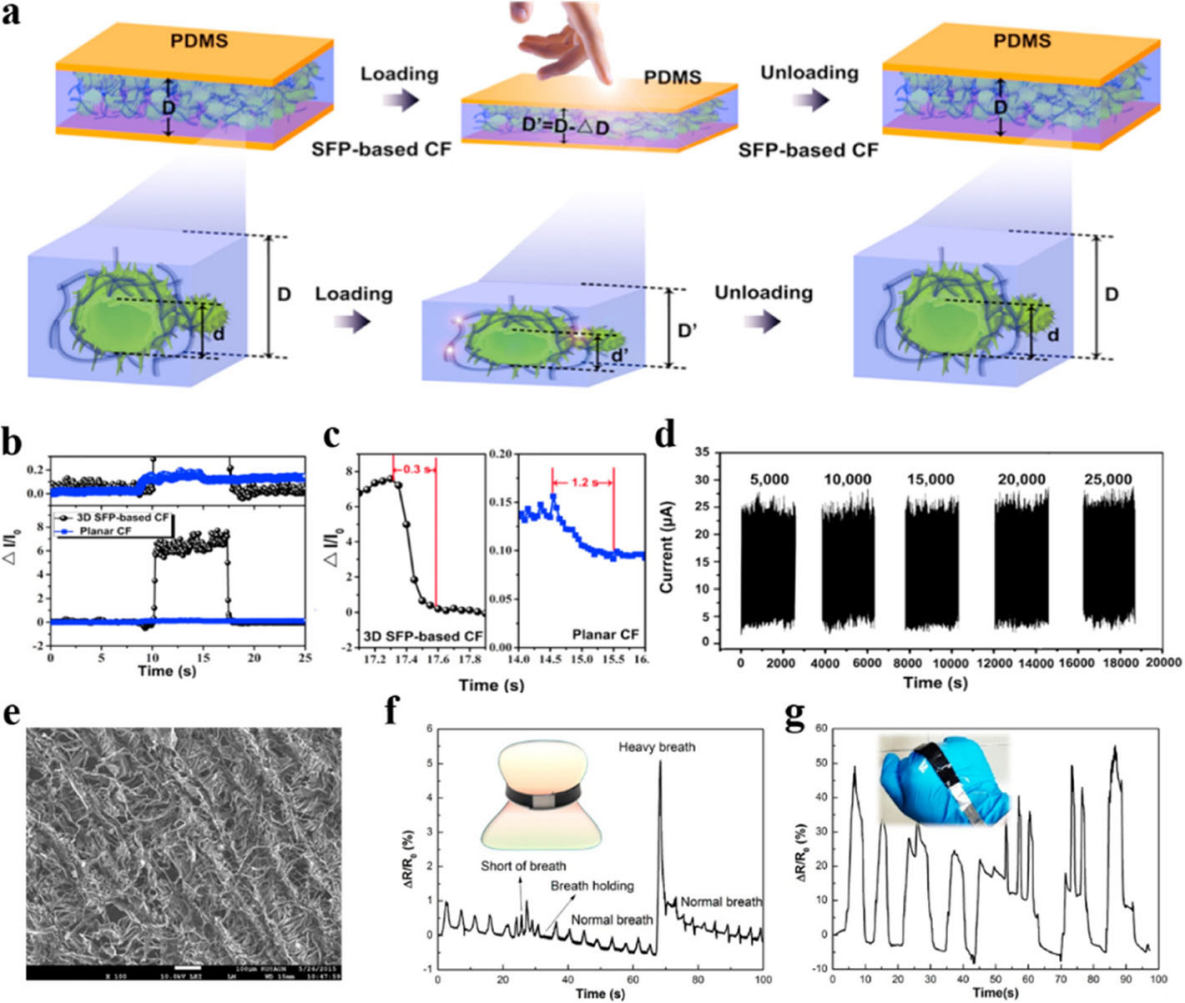
**a** Schematic of the mechanism of the sensor with hollow-sphere structure induced by sunflower pollen microcapsules under pressure. **b** Transient response of SFP-based composite film (CF) and planar CF under 600Pa pressure. **c** Relaxation time of the two related sensors. **d** Stability test of SFP-based CF at 80 Pa. Adapted with permission from ref. 3. Copyright 2017 Elsevier. **e** The SEM image of the converted carbon paper. **f**, **g** Monitoring the breath (**f**) and gesture (**g**) of an adult by the CP/PDMS sensor. Adapted with permission from ref. 63. Copyright 2017 American Chemical Society

Embedding conductive materials with porous structure into elastomer matrix to construct two- or three-dimensional conductive networks is another approach to achieve high sensitivity in resistive force sensors [7, 22, 23, 19, 24]. The deformation caused by external forces will change the spatial distribution density of conductive materials and thus change the resistance of the sensor. As displayed in Fig. 3a, Wang et al. [7] produced hollow-sphere conductive composites by combining sunflower pollen (SFP) microcapsules with multiwalled carbon nanotube (MWCNT), and then added them into PDMS to prepare a MWCNT/PDMS composite film. An E-skin device was fabricated by sandwiching this MWCNT/PDMS composite film between two conductive electrodes. As shown in Fig. 3b–d, compared with planar sensor, this hollow-sphere architecture introduced by pollen-based microcapsules enabled the sensor to show higher sensitivity, faster relaxation time, and very high stability. The sensor could simultaneously detect pressure and strain dynamically when attached to a human finger or a human throat. Li et al. [23] introduced a simple method to construct porous conductive networks by converting tissue paper into carbon paper (CP) via a high-temperature pyrolysis process. Figure 3e is the SEM image of the carbon paper. A highly sensitive strain sensor composed of the carbon paper and PDMS resin was successfully fabricated through a simple vacuum infusion process. The porous structure rendered the sensor ultra-high sensitivity to applied strain, almost one order of magnitude higher than that of traditional metallic sensor. As shown in Fig. 3f, 3, the CP/PDMS sensor demonstrates monitoring of the breath of an adult and the gesture of a human hand through integration with a belt and a glove respectively. Lee et al. [22] fabricated pressure-sensitive nanofibers with porous structure by using electrospinning process. The conducting nanomaterial (CNTs and graphene) was dispersed uniformly inside the nanofibers to improve the sensing ability. On account of the nanoporous structure, the resistive-type pressure sensor manufactured by using these composite nanofibers exhibited a high sensitivity for pressure-induced deformation and excellent conformability to three-dimensional structures.

### Capacitive Force Sensors

Capacitive sensors can response indicating changes in the external forces through changes in capacitance. A capacitor generally consists of a dielectric layer sandwiched by two conductive plates. The formula used to calculate the capacitance is $C=\frac{\varepsilon_0{\varepsilon}_rA}{d}$, where ε_0_ is the vacuum permittivity, ε_r_ is the relative permittivity of the dielectric, *A* is the effective overlap area of the two conductive plates, and *d* is the spacing between the two conductive plates. The electrodes of flexile capacitive force sensors usually use CNTs [25], Ag nanowires [26, 3], and conductive ionic materials [27]. Low modulus elastic materials including PDMS, SEBS, and Ecoflex are good candidates for dielectric layer.

**Fig. 4:**
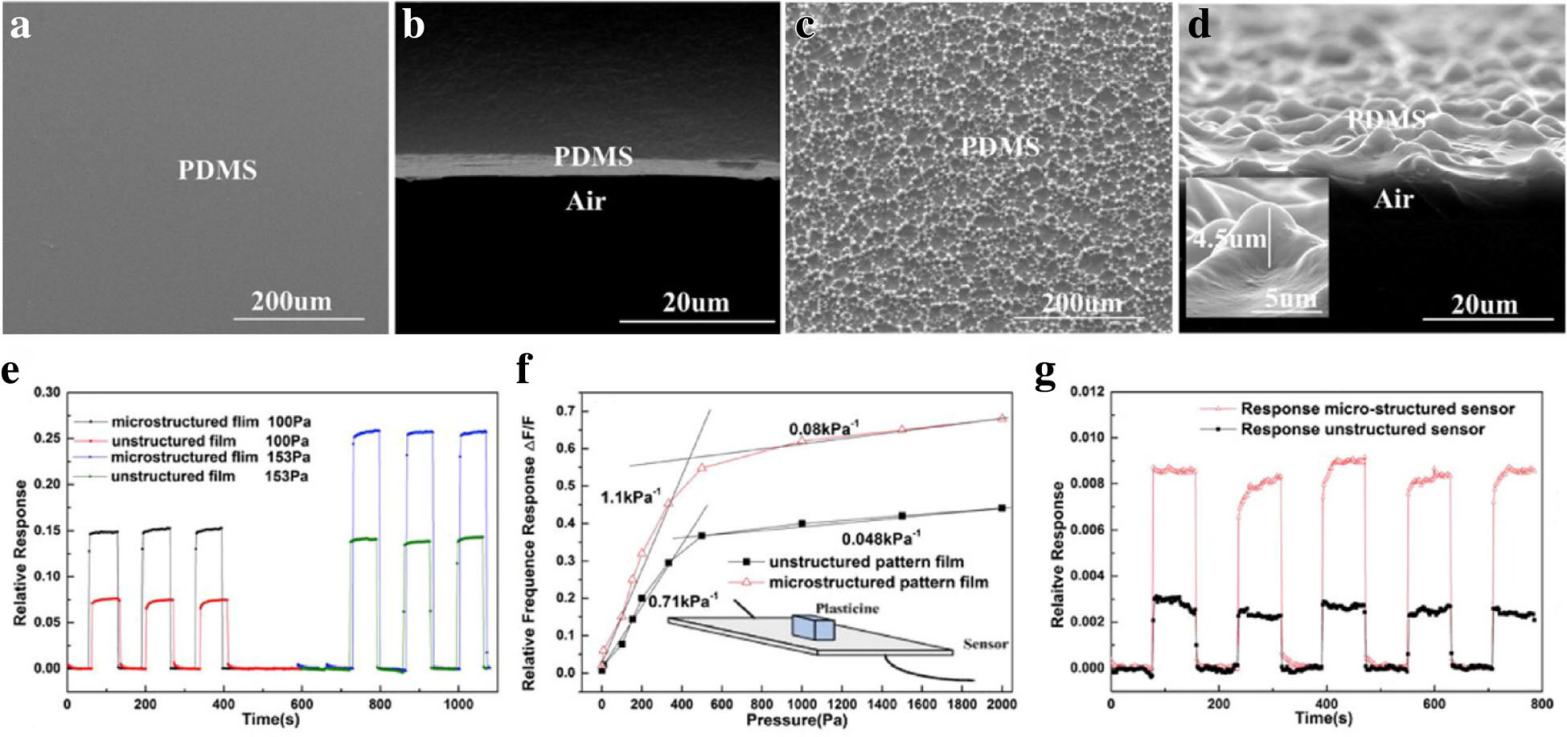
**a**–**d** SEM image of top view (**a**) and side view (**b**) of unstructured PDMS film, SEM image of top view (**c**), and side view (**d**) of microstructured PDMS film. **e** Comparison of the relative responses of the sensors with different structures. **f** Sensitivity testing of the two structured sensors. **g** The response of the two structured sensors under 1 Pa pressure. Adapted with permission from ref. 18. Copyright 2017 Elsevier

**Fig. 5:**
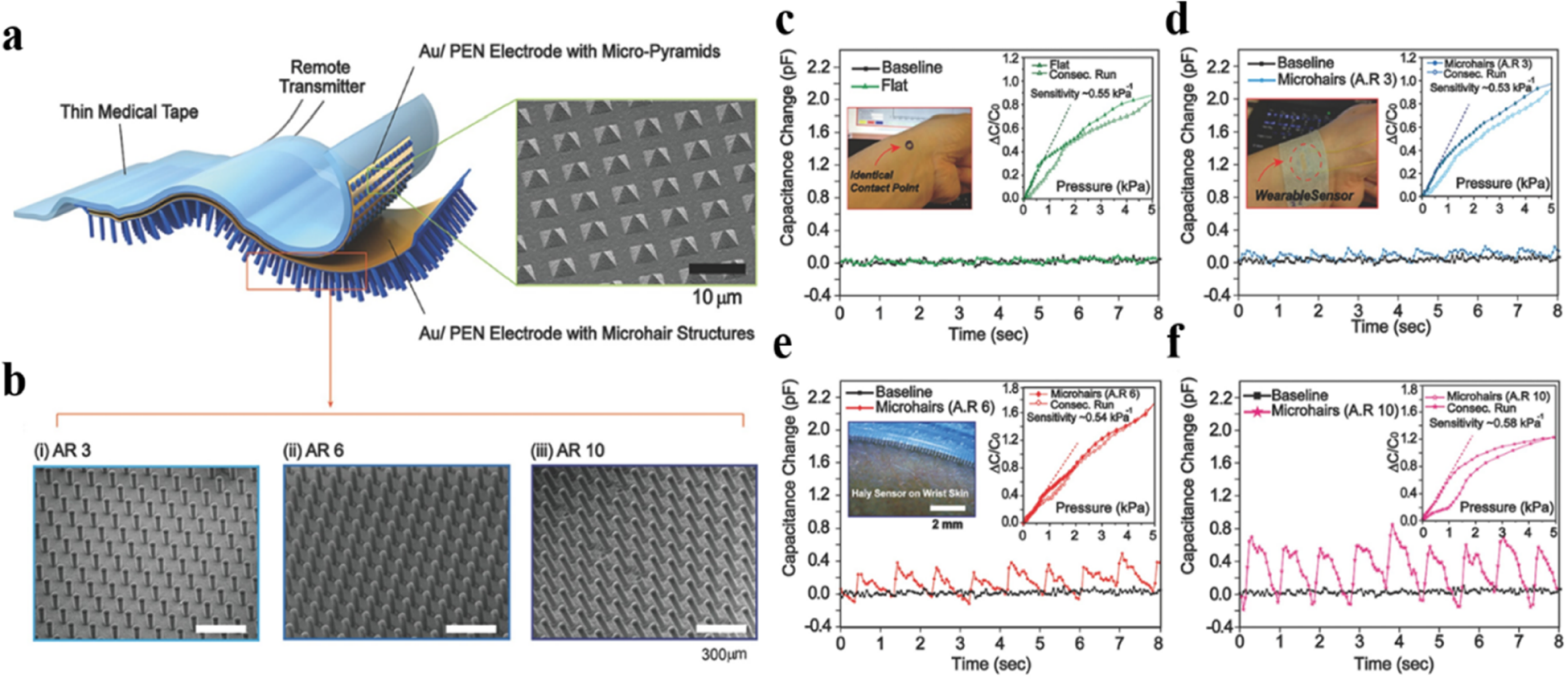
**a** Schematic diagram of the microhair-structured sensor. **b** SEM image of the microhair structure with different aspect ratios. **c**–**f** Radial artery test using four devices with different interface geometries: **c** flat surface, microhairy structure with aspect ratios of **d** 3, **e** 6, and **f** 10

The sensing ability of capacitive sensors can be significantly enhanced by microstructuring electrodes or dielectric layers [3, 2, 28]. As shown in Fig. 4a–d, Quan et al. [3] used matte surface glass as templates to prepare microstructured PDMS films as the electrode substrates for flexible capacitive sensors. They compared sensors with microstructured electrodes to those without one. The results in Fig. 4e–g demonstrated that sensors with microstructure exhibit higher sensitivity, lower detection limits, and a faster response time. Kang et al. [28] developed a high-performance capacitive pressure sensor based on a sponge-like porous dielectric layer. The sponge-like porous structure was achieved by coating PDMS on a silicon substrate stacked with polymer microbeads followed by dissolving the polymer microbeads. The porous PDMS film was then transferred to an ITO thin-film electrode, giving rise to a capacitive sensor with ultra-high sensitivity and high stability. The sensitivity of porous PDMS pressure sensors is more than eight times higher than that of sensors based on bare PDMS film. The reason for the better performance of the microstructured capacitive sensor can be attributed to the following two points. For one thing, structuring the elastomer electrode substrate or dielectric layer improves the compressibility of the device. For another, microarchitectures add air voids between the conductive plates of the capacitor in an orderly fashion, which makes the permittivity changeable under pressure. When an external force is applied to the sensor to cause deformation, the total volume of air voids in the dielectric layer decreases and the permittivity of air/elastomer hybrid dielectric layer increases, so that the rise in the capacitance value of capacitive sensors caused by two factors: the reduction in the plate spacing and the increase of permittivity. In addition, Pang et al. [2] developed a highly sensitive pressure sensor with a pyramidal-shaped PDMS dielectric layer and a microhair-structured interface, as displayed in Fig. 5a, b. Figure 5c–f compared radial artery test results by four sensors with different interface geometry, which revealed that the microhairy interface can obviously enhance the signal-to-noise ratio of capacitive pressure sensors.

To improve the sensitivity, integrating with organic field-effect transistor (OFET) is also a widely studied project for capacitive sensors. In OFET devices, the source-drain current is directly dependent on the gate dielectric capacitance. Schwartz et al. [29] reported highly sensitive OFET E-skin devices using microstructured PDMS film as the dielectric layer and a novel conjugated polymer, polyisoindigobithiophene-siloxane (PiI2T-Si) [30] as the semiconductor. The OFET device integrating a microstructured PDMS dielectric achieved ultrahigh sensitivity (8.4 kPa^−^1) in the low-pressure regime < 8 kPa as well as fast response time (< 10 ms). These superior capabilities demonstrated that such a device is promising in high-fidelity measurements of wrist pulse wave.

Compared with resistive sensors, capacitive sensors generally have higher sensitivity and lower detection limits. However, their poor linearity response and susceptibility to parasitic capacitance and fringing capacitance can be challenges in practical applications.

### Piezoelectric Force Sensors

Piezoelectric effect refers to the phenomenon that the mechanical stimuli deforms some anisotropic crystalline materials and causes the polarization of internal dipoles, leading to potential differences existing between the two opposing surfaces of the crystals. Due to the unique characteristics of piezoelectric materials, piezoelectric sensors with rapid response time are capable to measure high-frequency dynamic signals efficiently and are quite promising for self-powered devices.

**Fig. 6:**
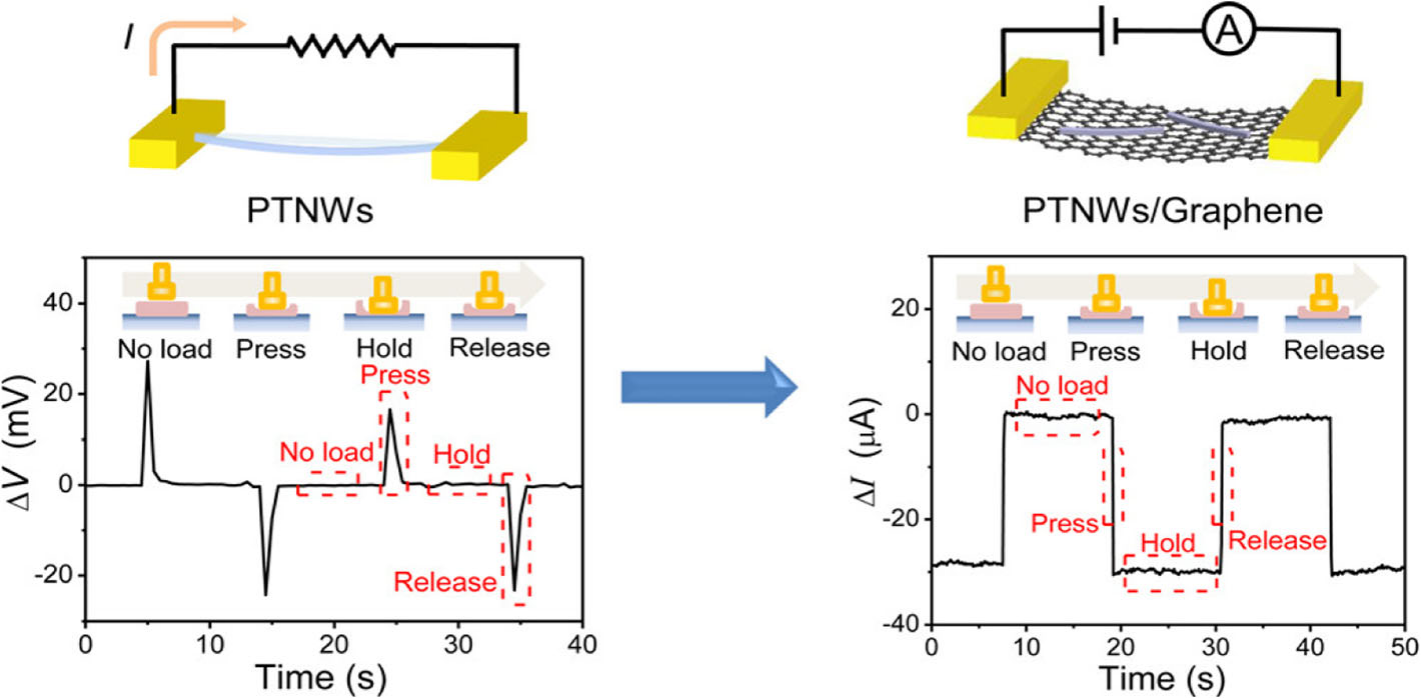
Pressure response of PTNW-based pressure sensor (left) and of a PTNWs/graphene transistor under a pressure pulse. Adapted with permission from ref. 25. Copyright 2017 American Chemical Society

Piezoelectric materials commonly used in flexible sensors include P(VDF-TrFE) [31, 32], ZnO [33], PbTiO3 [34], and PZT [35, 36] etc. P(VDF-TrFE) is one of the most favorite materials for flexible piezoelectric sensors because of its flexibility, simple fabrication process, remarkable stability, and large piezoelectric coefficient. Persano et al. [31] reported a flexible piezoelectric sensor based on aligned P(VDF-TrFE) fiber arrays prepared by electrospinning. This simple pressure sensor exhibits excellent sensing performance even in the extremely small pressure regime (around 0.1 Pa). The results suggested extraordinary application potential in human motion detection and robotic electronics. Although inorganic materials are short of flexibility, many nanoscale inorganic materials and polymer–ceramics nanocomposites (such as ZnO NWs [33], PZT nanoribbons [35] and nanosheets [36], and P(VDF-TrFE)/BaTiO_3_ nanocomposite [4]) can exhibit a certain degree of flexibility. Shin et al. [33] employed lithium (Li)-doped ZnO NWs packed into PDMS as sensing element. The piezoelectric output voltage of the Li-doped ZnO NW–PDMS composites was a function of applied force and frequency. The fabricated devices were capable of providing instantaneous information of human motions, which is of great significance for the application of electronic skin devices in human-activity monitoring. The piezoelectric sensors are particularly useful for the detection of dynamic physical stimuli but do not perform well in measurement of static signals. This is because the voltage signal generated by piezoelectric materials will only appear at the moment when pressure is applied or withdrawn. To solve this problem, Chen et al. [34] reported a flexible piezoelectric pressure sensor for static measurement based on PbTiO_3_ nanowires (PTNWs)/graphene heterostructure. In this device, the polarization charges induced by the strain in PTNWs acted as charged impurities in graphene and affect its carrier mobility. The working mechanism is that the polarization charges in PTNWs increased the scatterings of carriers in graphene thus resulting in decreased carrier mobility. Based on the aforementioned mechanism, as shown in Fig. 6, this heterostructure sensor possessed higher sensitivity than intrinsic CVD-grown graphene pressure sensors [37, 38] and was capable of measuring static mechanical signals.

## Flexible Temperature Sensors

Temperature detection is an important part of sensing devices. Body temperature can reflect people’s physical condition to a large content. The body-core temperature of healthy people is relatively constant, generally contained between 36.2~37.2 °C. It is independent on the environment, while the shell temperature can be affected by both physical conditions and ambient temperature. Abnormal changes in body temperature usually indicate poor health. For example, an increased body-temperature is the symptom of fever or infection, while a degraded body temperature probably signifies anemia. For real-time detection of temperature in E-skin devices, many types of flexible temperature sensors have been developed.

### Resistive Temperature Sensors

Detecting temperature through changes in the resistance of sensitive materials is the most commonly used method for temperature measurement in skin-like electronics devices. Temperature coefficient of resistance (TCR) is an important indicator of the sensitivity of resistive temperature sensors. It is defined as the relative variation of resistance when the temperature changes by 1 °C. Various resistive temperature sensors have been reported using pure metal elements (Pt, Au, Cu) [39–42], metal oxide particles [43], carbon nanotube (CNT) polymer composites [8, 9], and graphene [44, 45] as sensitive materials.

**Fig. 7:**
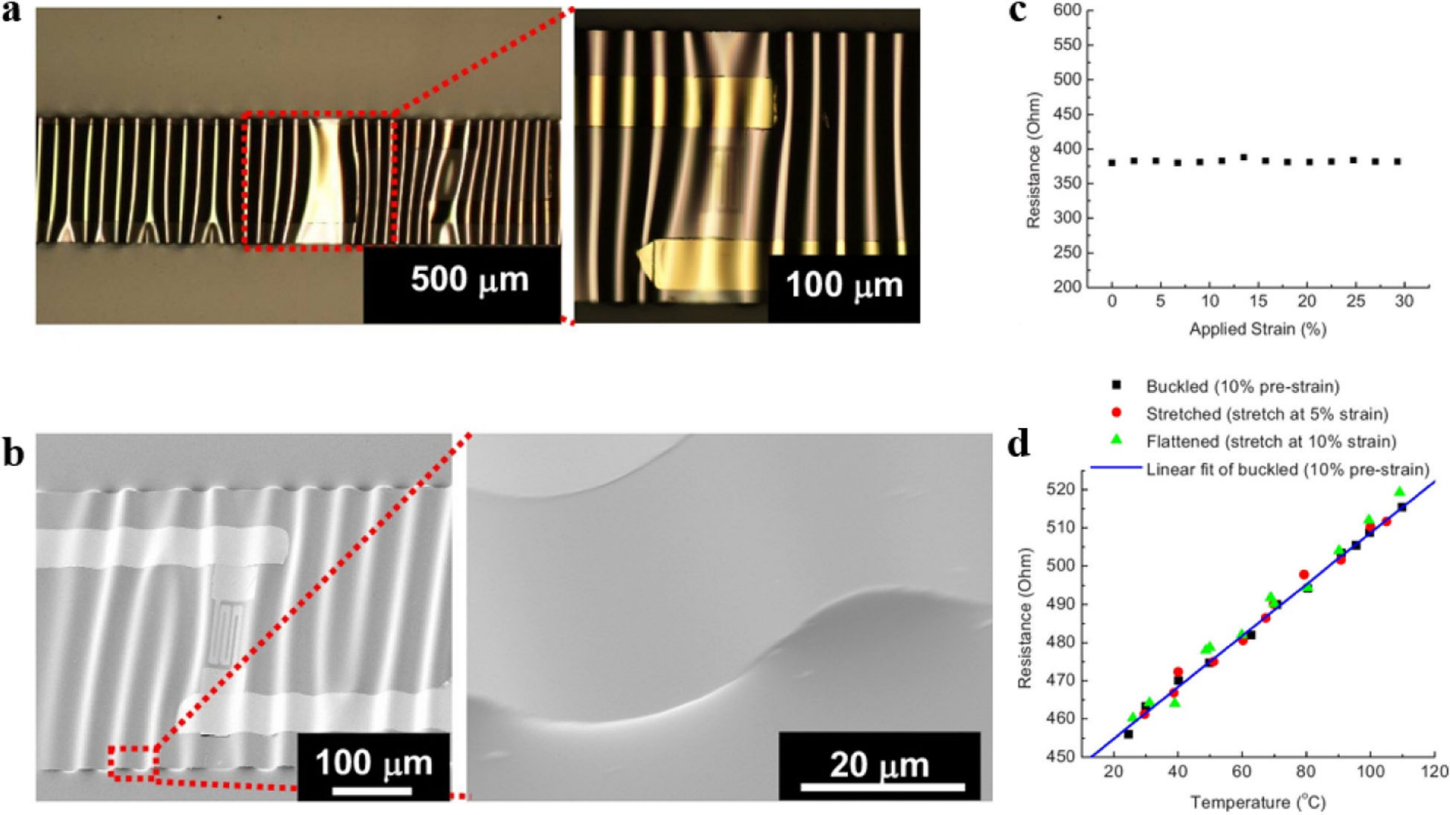
**a** Schematic of the stretchable sensors with periodically wavy patterns. **b** SEM of the stretchable temperature sensor. **c** Changes in the sensor’s resistance value when the sensor strain is continuously stretched from 2.25 to 30%. **d** The relationship between resistance and temperature of a stretchable sensor with strains of 0%, 5%, and 10%. Adapted with permission from ref. 29. Copyright 2009 AIP Publishing

Metals have been used for temperature detection for a long time due to their temperature sensitivity. The detection mechanism can be explained by the fact that the rise in temperature enhances the thermal vibration of the lattice, resulting in intensified scattering of the electron wave, thus increasing the resistivity. Traditional metal-based temperature sensors provide limited stretchability or bendability. Structure engineering, such as wrinkle buckling, in-line horseshoe-like structure, and rigid-island design [39, 41, 46], has been certified an effective way to overcome the limitations. As displayed in Fig. 7a, b, Yu et al. [39] developed a stretchable temperature sensor based on corrugated thin film sensing elements on elastic substrate. The sensor was fabricated by sputtering deposition of a thin Cr/Au film (5 nm/20 nm) on a pre-stretched 30% flexible substrate. As shown in Fig. 7c, d, the periodical wavy geometry formed by releasing pre-strain allows the device to stretch up to 30% mechanical strain with unchanged performance. Webb et al. [41] reported an ultrathin, compliant skin-like temperature sensor array using thin (50 nm), narrow (20 μm), gold thin film in a shape of serpentine prepared by microlithographic techniques. When implemented with advanced modeling and analysis techniques, the stretchable electronic systems were capable of noninvasive mapping of shell temperature in millikelvin accuracy.

**Fig. 8:**
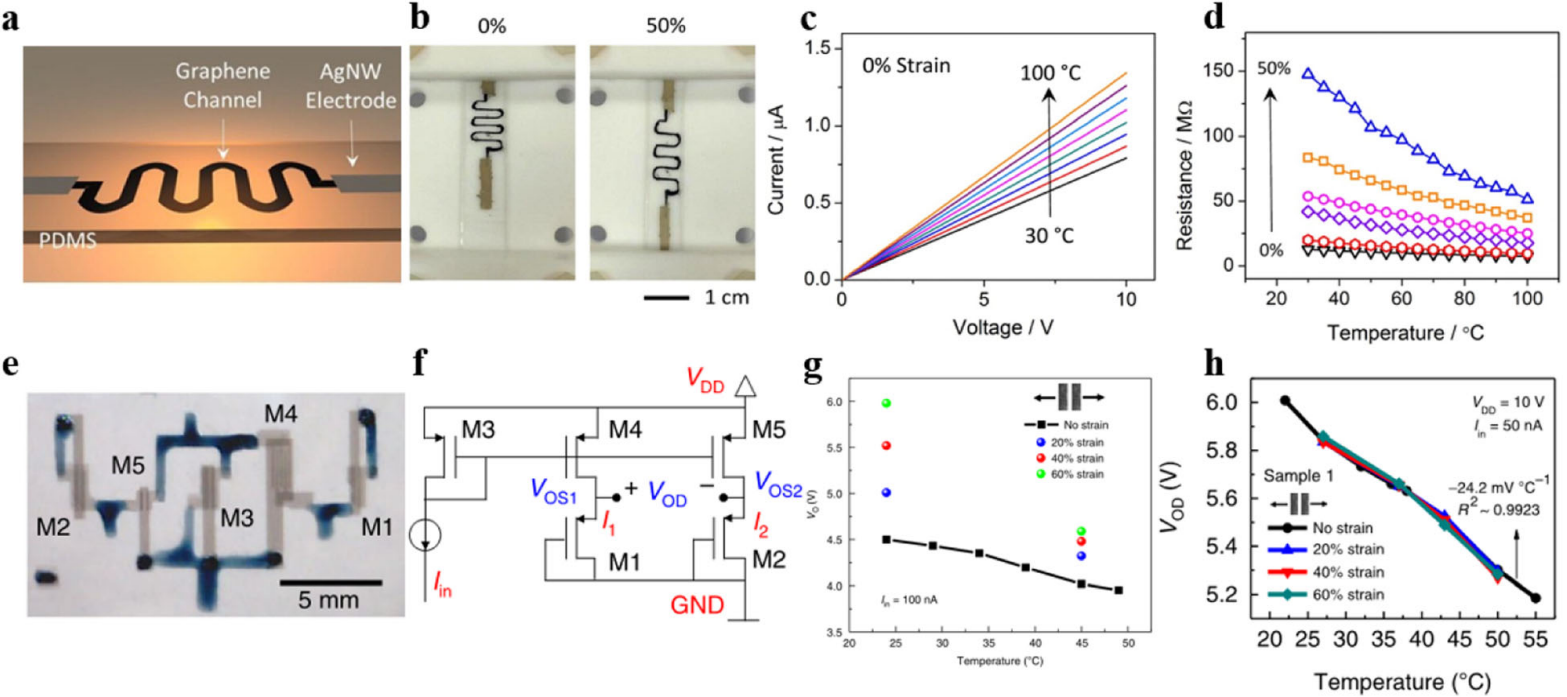
**a** Schematic diagram of the stretchable of the graphene thermistors. **b** Image of the graphene thermistor at 0% and 50% strain. **c** Resistance variation with temperature. **d** Resistance variation with temperature within 0–50% strains. Adapted with permission from ref. 35. Copyright 2015 American Chemical Society. **e** Optical micrograph of a stretchable temperature-sensing circuit consisting of five TFTs. **f** Circuit schematic of the static differential sensing approach. **g** Temperature sensing performance of a single TFT. **h** Temperature sensing performance of a stretchable static differential circuit sensing device. Adapted with permission from ref. 39. Copyright 2018 Springer Nature

Above-mentioned works have effectively improved the flexibility of metal-based temperature sensors, but the structure engineering methods used in those devices limited the stretchability to 25–30%. To further break the tensile limit of flexible temperature sensors, the use of inherently stretchable materials is required. Harada and co-workers [8, 9] introduced flexible temperature sensors based on poly(3,4-ethylenedioxythiophene)-poly (styrene sulfonate) (PEDOT: PSS)-CNT composite film prepared by a printing process. The sensitivity of PEDOT: PSS-CNT mixed temperature sensor is from 0.25 to 0.63%/°C in different composite ratios of the CNT paste and PEDOT: PSS solution, which is better than metal-based temperature sensors [39–42]. As shown in Fig. 8a, b, Yan et al. [45] developed a stretchable graphene-based thermistor by using a lithographic filtration method to prepare graphene detection channel with microporous structure. The device indicates high intrinsic stretchability up to 50% and its TCR can be effectively tuned by mechanical strain, as exhibited in Fig. 8c, d. However, strain dependence is not ideal for wearable sensing because stretching or twisting the sensor can alter the resistance of the thermistor. In the case of sensor deformation, it is not possible to read strain and temperature values from a single numerical signal. It is still a challenge to avoid the influence of strain effects on temperature sensing in thermistors prepared with inherently stretchable materials. In order to obtain high stretchability and strain adaptability simultaneously, Zhu et al. [47] reported a temperature sensor based on CNT transistors with strain suppression capability by designing differential circuits (the circuit diagram was shown in Fig. 8e, f). A single stretchable thin-film transistor with supramolecular-polymer-sorted SWCNTs patterned as the semiconductor channel was fabricated as a temperature detection device. Dense unsorted SWCNT networks and a nonpolar SEBS thin film were used as source–drain and gate electrodes and the gate dielectric, respectively. The main mechanism can be attributed to the temperature dependence of charge transport in the semiconducting SWCNT network [48]. The strain-induced threshold voltage shift was nullified by employing the static differential circuit configuration, as shown in Fig. 8g, h. The differential output voltage (V_OD_) can thus be suppressed as long as they match between the two branches.

It is worth mentioning that such TFT structure device has been proven by other researchers to significantly improve the sensitivity of temperature sensors. Trung et al. [44] fabricated stretchable resistive and gated temperature sensors for wearable electronics and compared the performance differences between the two types of sensors. The temperature sensing layer was a composite conductive material formed by inserting temperature-responsive R-GO nanosheets into an elastomeric PU matrix. According to their test results, gated devices achieved higher temperature sensitivity (1.34% per °C) than resistive devices (0.9% per °C).

### Pyroelectric Temperature Sensors

A variation of temperature will change the remnant polarization of pyroelectric materials thus generating opposite bound charges on both surfaces of the crystal. Materials that have been found to exhibit pyroelectricity include different ceramics (PZT, LiTaO_3_, LiNbO_3_) and polymer (PVDF, P(VDF-TrFE)) [49–53]. A lot of pyroelectric devices have been fabricated on rigid substrate and widely used in missile detection, fire alarm, and other fields. Nevertheless, flexible pyroelectric devices still need to be explored. In particular, P(VDF-TrFE) is ideal for temperature sensing applications in flexible electronics. Tien et al. [51] directly used a highly crystalline β-phase P(VDF-TrFE) material with extremely large remnant polarization as gate insulator in an OTFT structure for temperature sensing. The remnant polarization inside the P(VDF-TrFE) can change with temperature, causing a change in the density the holes accumulated at the interface between the semiconductor channel and P(VDF-TrFE). Therefore, the source-drain current increases as the increase of temperature. The linear response of the device in a certain temperature range and its simple fabrication process suggest its potential application in flexible temperature sensors. However, for (P(VDF-TrFE)), the pyroelectric effect is indistinguishable from the piezoelectric effect, which means that mechanical deformation will interfere with temperature detection. To decouple strain-induced interference from temperature effect, Tien et al. [54] developed flexible pyroelectric OFET devices with piezo- and pyroelectric nanocomposite gate dielectrics formed by a mixture of (P(VDF-TrFE)) and BaTiO_3_ nanoparticles as well as piezo- and thermoresistive organic semiconductor channel(pentacene). The fabricated devices can extract effects from the target sensing signals successfully while the flexible sensor is under multiple stimuli because the two chosen materials were able to respond to strain and temperature in a disproportionate manner simultaneously. This approach is able to distinguish the temperature effects from strain for flexible pyroelectric sensors.

## Flexible Physiological Biochemical Sensors

In order to understand all aspects of human health, various physiological biochemical sensors have been developed for analysis of vital biochemical signs, such as blood glucose [55, 56, 57, 58] and body fluids (sweat, interstitial fluids, saliva, and tears) [59, 60, 61]. Flexible biochemical sensors typically adopt chemical methods to detect the composition and amount of a biological substance. The chemical reaction between the sensing material and the target detection substance changes the electrical properties of the sensor, therefore the physiological health information can be obtained by analyzing the electrical parameters of the sensor.

**Fig. 9:**
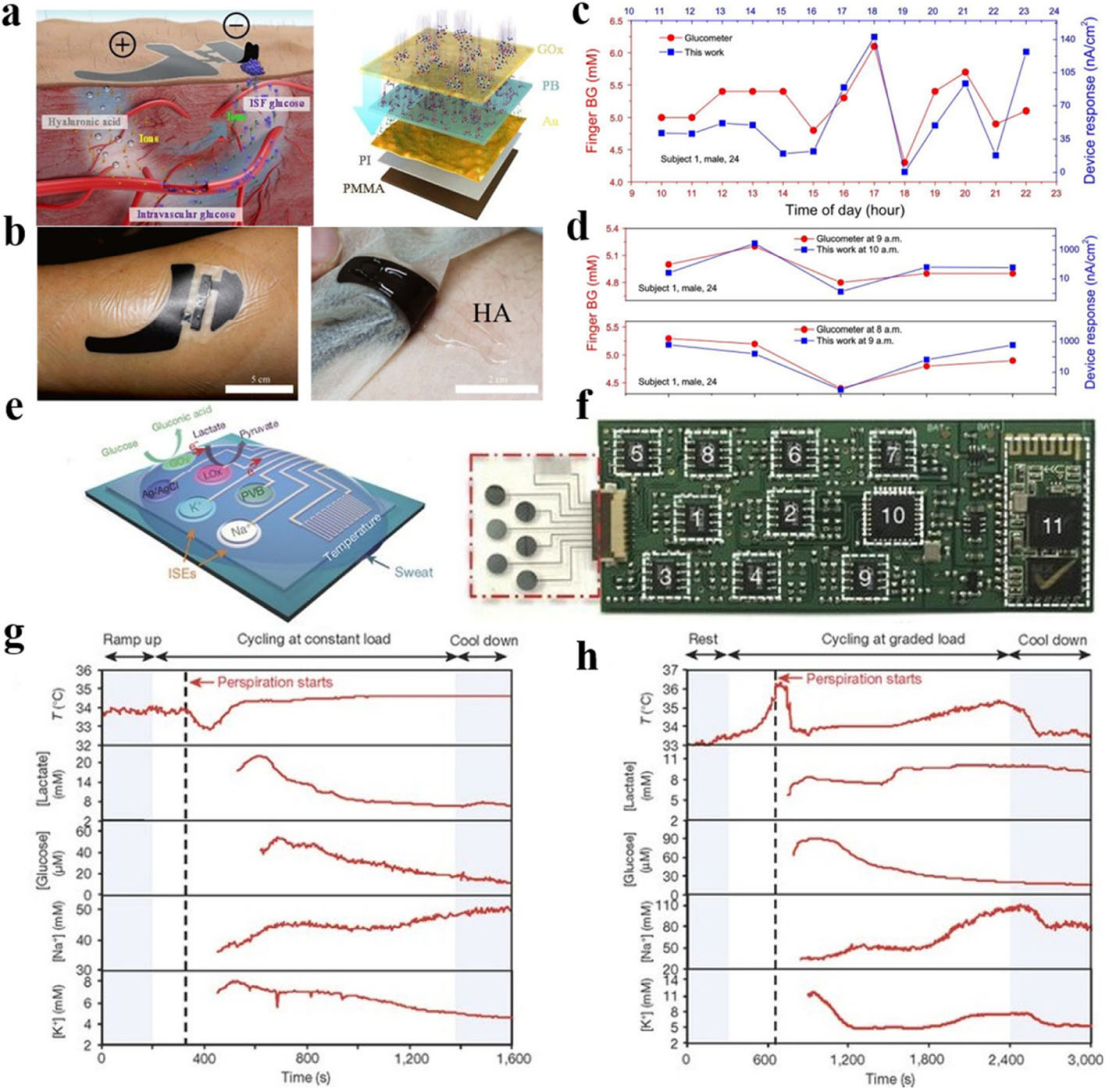
**a** Schematic of the ETCs (left) and the biosensor multilayers (right). **b** A biosensor attached to skin surface for glucose monitoring. **c** Results of glucose monitoring in one day by a glucometer and a biosensor. **d** Results of glucose monitoring in 5 days by a glucometer and a biosensor. Adapted with permission from ref. 48. Copyright 2017 American Association for the Advancement of Science. **e** Schematic of the sensor system for multiplexed sweat analysis. **f** Photograph of a flexible integrated sensing device. **g** The result of sweat analysis by wearing the sensor on the forehead of the subject. **h** The result of sweat analysis by wearing the sensor on the forehead of another subject. Adapted with permission from ref. 54. Copyright 2016 Springer Nature

Continuous measurement of glucose is vital to maintain the health and quality of life of diabetics. Commercially available products for glucose detection are performed by invasive lancet approaches that requires sampling the patient’s blood, leading to pain to the patient. New electronics fabrication techniques on flexible substrates have been developed to enable noninvasive wearable glucose monitoring. Chen et al. [55] developed a skin-like biosensor for noninvasive blood glucose monitoring via electrochemical channels. The detection mechanism and structure of this sensor are shown in Fig. 9a, b. A paper battery was attached to the skin to produce subcutaneous electrochemical twin channels (ETCs), through which more intravascular blood glucose was expelled from the blood vessel and transported to the skin surface. The outward-transported glucose thus can be measured easily by a glucose oxidase (GOx) immobilization layer. The experimental test results are shown in Fig. 9c, d. As can be seen from the figure, the monitoring results of the biosensor are in good agreement with the results of the commercial glucometer. Besides glucose monitoring, sweat analysis can be important in facilitating insight into an individual’s heath state. For example, sweat glucose is metabolically related to blood glucose and low electrolyte levels in sweat may be a sign of dehydration. Gao et al. [61] presented a highly integrated wearable sensing system for multiplexed in situ sweat analysis. As shown in Fig. 9e, f, the sensing system composed of four different sensing elements for simultaneous and selective screening of a panel of biomarkers in sweat—sodium (Na+), potassium (K+), sweat glucose, and sweat lactate. They also exploited a flexible printed circuit board (FPCB) to realize the conditioning, processing, and wireless transmission of critical signals. According to the test results in Fig. 9, it can be seen that the wearable system can be used to measure the detailed sweat characteristics of a human subject and to evaluate the physiological state of the object in real time.

## Multifunctional Sensors

**Fig. 10:**
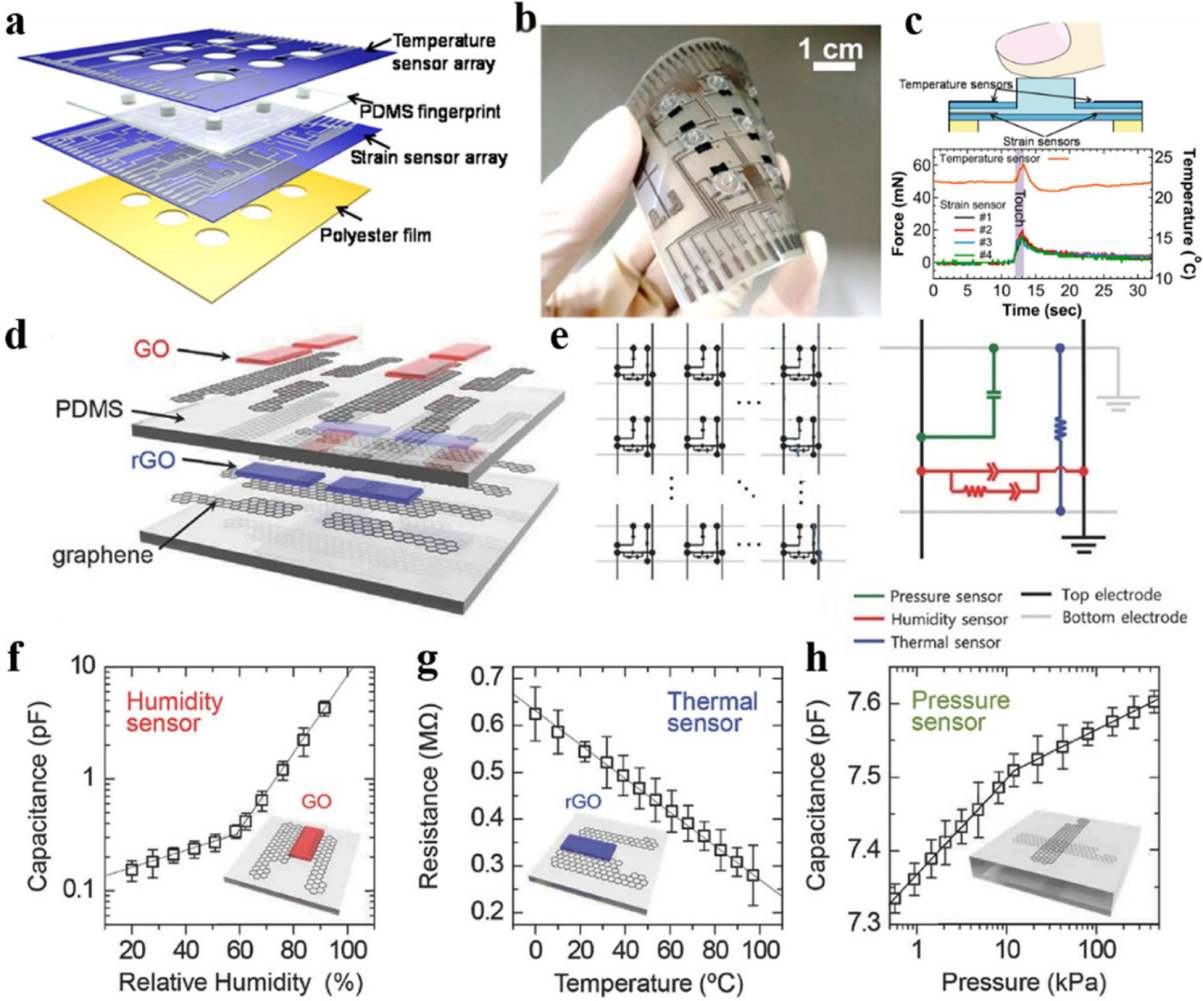
**a** Schematic for the structure of multilayer sensor. **b** Picture of a 3 × 3 sensor array. **c** schematic and measurement results of the multifunctional sensor when touching a fingerprint-like structure with a human finger. Adapted with permission from ref. 4. Copyright 2014 American Chemical Society. **d** Schematic diagram of the multimodal e-skin sensor. **e** Circuit diagram of the sensor matrix. **f** Performance of the humidity sensor based on GO. **g** Performance of the temperature sensor based on rGO. **h** Performance of the pressure sensor based on PDMS. Adapted with permission from ref. 55. Copyright 2016 John Wiley and Sons

Integrating multifunctional sensing components into one device is an important advance in wearable electronics. Future wearable electronics should enable to integrate the function of detecting multiple signals such as strain, pressure, temperature, humidity, gas [8, 9, 62, 63], and so on into a single device to provide more comprehensive human health and environmental information. Laminating multiple layers of thin film e-skin device with different sensing functions together is the major method to prepare multifunctional sensors. Harada et al. [8] fabricated a triaxial tactile sensor and temperature sensor array to simultaneously detect the tactile forces, slip forces, and temperature by using a printing manufacturing technique. Four strain sensors printed by a screen printer were designed with a PDMS fingerprint for a pixel, as shown in Fig. 10a, b. Three-axis force directions can be detected by characterizing the strain distribution at the four integrated force sensors with a finite element method (FEM). Figure 10c shows the measurement results of the multifunctional sensor when touching a fingerprint-like structure with a human finger. The integrated strain/temperature sensing array for e-skin application show good performance in imitating human skin. Ho et al. [62] developed a multimodal all graphene e-skin sensor matrix. Three different sensors—humidity, thermal, and pressure sensors—were included in this matrix. Sprayed graphene oxide (GO) and reduced graphene oxide (rGO) were used as active sensing materials for the humidity and temperature sensors, respectively. Whereas the top PDMS substrate sandwiched between two CVD-graphene electrodes acted as the capacitive strain sensor, as displayed in Fig. 10d, e. The three sensors were integrated into a single unit through a simple lamination process. As can be seen from the test results in Fig. 10f–h, each sensor is sensitive to its associated external stimulus, but not affected by the other two stimuli. These results indicate that the E-skin device offers unique opportunities for healthcare applications in the future.

## Functional Modules of Wearable Electronics

In order to develop highly integrated wearable system for applications in health monitoring, physical state assessment, and telemedicine, researchers have tried various manufacturing processes and device structures to combine different functions together. Self-powered modules working continuously without external power sources should be an integral part of future wearable electronics. In addition, for real-life application of wearable electronics in monitoring critical health information, a wireless digital system for processing and transmitting signals over long distances is necessary.

**Fig. 11:**
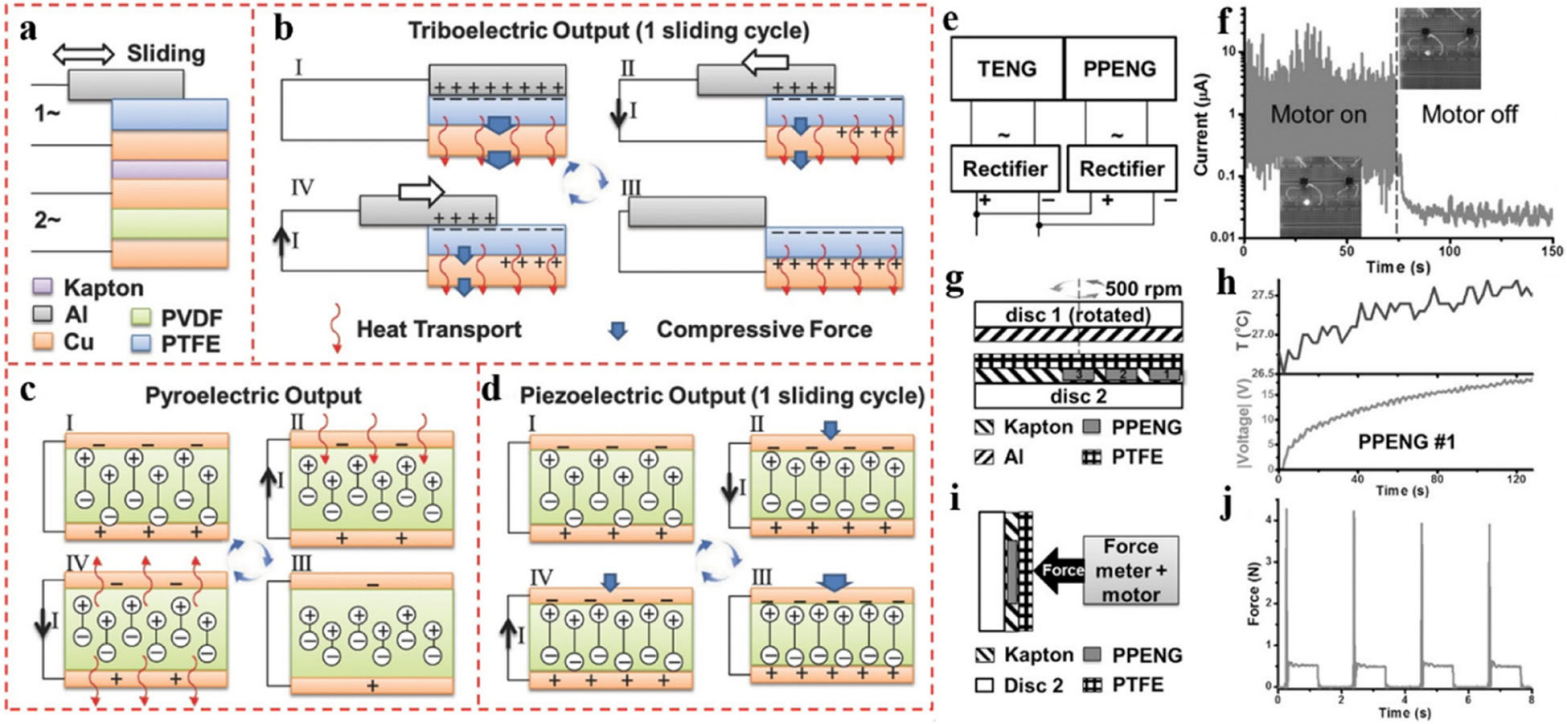
**a**–**d** The structure and working principle of the tribo-, pyro-, and piezoelectric hybrid cell. **e** The circuit that hybridizes TENG and PPENG outputs. **f** The LED was lighted by the hybridized output current. **g** The schematic diagram of the structure used to demonstrate the temperature sensing. **h** The voltage and the temperature variation of the PPENG. **i** The schematic diagram of the measurement setup. **j** A force of approximately 0.5 N applied to the surface. Adapted with permission from ref. 56. Copyright 2015 John Wiley and Sons

To realize independent operation of wearable sensors, nanogenerators based on piezoelectric, pyroelectric, and triboelectric effects have been developed to incorporate into wearable systems [64–67]. Nanogenerators are able to harvest mechanical energy or thermal energy from human activities to power wearable devices. Zi et al. [64] developed a tribo-, pyro-, and piezoelectric hybrid cell that is composed of a sliding mode triboelectric nanogenerator (TENG) and a pyroelectric-piezoelectric nanogenerator (PPENG) for self-powered sensing. The structure and working principle of the hybrid cell are shown in Fig. 11a–d. The TENG, fabricated with a piece of aluminum foil as the sliding layer and a piece of polytetrafluoroethylene (PTFE) film deposited on Cu electrode as the static layer, is able to harvest the sliding mechanical energy. The PPENG was fabricated by depositing a piece of PVDF with Cu electrodes on both sides to harvest the thermal energy generated by friction and the mechanical energy generated by the normal force. As can be seen from Fig. 11e–j, the hybrid cell is demonstrated as an efficient power source that can drive the LED with extended lighting time, and a versatile self-powered sensor for detecting both the subtle temperature alteration and strain on the surface of human skin. Nevertheless, the rapid development of flexible electronics places higher demands on corresponding power devices, which should be comparably flexible or stretchable. Pu et al. [65] reported a soft skin-like triboelectric nanogenerator that achieves ultrahigh stretchability (maximum stretch up to 12.6 or strain of 1160%) and high degree of transparency (96.2%) by using PDMS or LED as the elastomer electrification layer and PAAm-LiCl hydrogel as the electrode. This skin-like generator is capable of outputting an open circuit voltage of up to 145 V and an instantaneous power density of 35 mW m^−2^ through harvesting biomechanical energy. Meanwhile, the TENG-based electronic skin can serve as a tactile sensor to sense pressure and achieved a sensitivity of 0.013 kPa^−1^. The development of self-powered, wearable platforms has opened up opportunities for many potential applications including soft robots, smart artificial e-skins, wearable electronics, etc. However, there are still limitations of flexible energy harvesting devices because the power generation of nanogenerators that have been reported so far cannot meet the needs of practical applications.

The integration of flexible sensors with information processing system is the next frontier for wearable electronics. Current research on flexible electronics mainly focused on the fabrication and optimization of sensing elements, while the research on flexible electronic circuits for information processing is relatively limited. The delivery and processing of human health information collected by the sensor still needs to be done by a computer. Wireless transmission of sensor data that has been reported so far is principally realized by combining a flexible sensor with a rigid silicon-based digital circuit technology. Pang et al. [2] built a custom wireless measurement system based on an XBee Series 2 radio module integrating to a programmed Arduino microcontroller. This system allows the sensor data to be wirelessly transmitted to a computer but is relatively bulky and not portable. Gao et al. [61] devise a multiplexed sensing system that integrated the functions of signal conditioning, processing, and wireless transmission by merging commercially available technologies of consolidating integrated circuits on a flexible printed circuit board (FPCB), with flexible sensor technologies fabricated on elastic substrates. The introduction of FPCB technology bridges the technological gap between signal conditioning, processing, and wireless transmission in wearable sensors to some extent, but the flexibility and comfort of the system still do not meet the requirement of next-generation wearable electronics. Realizing skin electronics rely on the development of intrinsically stretchable circuits [68].

## Conclusions and Outlook

In past several years, the rapid development of wearable electronics attracts extensive attention. Researchers have made many fruitful attempts and achieved good results in developing wearable electronics with high sensitivity, flexibility, and stability. This review analyzed recent research strategy and advancements in wearable electronics for human health detection from the aspects of force sensors, temperature sensors, physiological biochemical sensors, multi-functional sensor, and other functional modules applied in flexible electronics. The successful fabrication of flexible sensing devices with high sensitivity, low-cost, portability, and long-term stability indicates that flexible and wearable electronics will definitely become the mainstream in the field of medical care in the future. However, there are certain challenges remaining for practical applications of current wearable sensors in real life.

1. Wearable electronic devices should be able to clearly identify the deformations caused by pulse, muscle movements, and external contact. While most of the flexible force sensors that have been reported so far cannot accurately identify the source and direction of external forces.
2. In terms of temperature sensors, it is still difficult to achieve high stretchability, sensitivity, and strain adaptability simultaneously. Improving the sensing performance and eliminating the influence of the elastic deformation of the sensor on temperature detection remain important research topics.
3. The detection accuracy of flexible physiological biochemical sensors is insufficient compared to traditional medical devices. Besides, most of the valuable physiological health information needs to be extracted from internal secretions. More biophilic implantable materials should be taken into consideration for the development of biochemical sensors to extract information from blood and muscles.
4. Multifunctional sensors should be able to simultaneously detect pressure, stress, temperature, and other different signals such as humidity and gas atmosphere and avoid crosstalk between them. The realization of multifunctional sensors requires further development of new materials, nanotechnology, and device structure design.
5. Processing the data in situ and transmitting them in real time are also essential parts of future wearable electronics. It is quite challenging to integrate multiple functional modules into a complete wearable system so that it can fully meet the requirements of practical applications.

## Data Availability

Not applicable.

## Abbreviations

Au: Aurum
Cu: Cuprum
CVD: Chemical vapor deposition
LED: Light-emitting diode
NW: Nanowire
OFET: Organic field-effect transistor
P(VDF-TrFE): Poly(vinylidenefluoride-tirfluoroethylene)
PAAm: Polyacrylamide
PbTiO_3_: Lead titanate
PDMS: Polydimethylsiloxane
Pt: Platinum
PU: Polyurethane
PZT: Lead zirconate titanate
SEBS: Styrene-ethylene-butylene-styrene block copolymer
VHB: Very high bond
ZnO: Zinc oxide
